# Adhesion performance of a universal adhesive in the root canal: Effect of etch-and-rinse vs. self-etch mode

**DOI:** 10.1371/journal.pone.0195367

**Published:** 2018-04-09

**Authors:** Fereshteh Shafiei, Pourya Mohammadparast, Zahra Jowkar

**Affiliations:** 1 Oral and Dental Disease Research Center, Department of Operative Dentistry, School of Dentistry, Shiraz University of Medical Sciences, Shiraz, Iran; 2 Department of Operative Dentistry, School of Dentistry, Shiraz University of Medical Sciences, Shiraz, Iran; Institute of Materials Science, GERMANY

## Abstract

**Purpose:**

Universal adhesives are new systems that can be used in etch-and-rinse (ER) and self-etch (SE) modes. This in vitro study evaluated the bonding performance of a universal adhesive in ER mode and SE mode with two irrigants for luting fiber posts in the root canal.

**Materials and methods:**

After separation of the roots from the crowns of 56 maxillary central incisors and endodontic treatment, 10-mm post space was prepared. The roots were divided into seven groups according to irrigant/adhesive protocol used for cementation of posts: 1) sodium hypochlorite (NaOCl) irrigant + acid etching + One-Step Plus, 2) NaOCl + Clearfil SE Bond (CSE) and 3) EDTA + CSE as controls; 4) NaOCl + All-Bond Universal (AB) in ER mode, 5) NaOCl + AB in SE mode, 6) EDTA + AB in SE mode, 7) distilled water + AB in SE mode. Posts were luted using Duo-link. The bonded roots were sectioned into microslices. After push-out bond strength (PBS) testing, data in MPa were analyzed with two-way ANOVA and Tukey test (α = 0.05).

**Results:**

PBS was significantly affected by irrigation/adhesive protocol and root region (P<0.05), with no significant interaction of these factors. PBS of ABU in ER mode with NaOCl and in SE mode with NaOCl or EDTA was comparable to that in the respective controls. The highest and lowest PBSs were recorded for ABU in the SE mode with EDTA (15.38 ± 4) and NaOCl (10.17 ± 3.5), respectively. PBS of AB in ER and SE modes was similar when distilled water was used in the SE mode.

**Conclusion:**

Adhesive performance of AB in the ER mode was comparable to or different from the SE mode, depending on the irrigant used to prepare post space in SE approach. AB could behave as a reliable bonding for post cementation.

## Introduction

Use of fiber-reinforced composite posts (FRC) has increased in recent years because they provide esthetic and support for the restoration of endodontically treated teeth [1]. FRC posts with the elastic modulus similar to that of dentin uniformly distribute stresses in the root canal, avoiding stress concentration. This, along with bonding ability to root canal dentin through adhesive/resin cement, results in reduced risk of vertical fracture [2, 3]. Furthermore, they minimize tooth structure preparation, enhance appearance and provide better light transmission to the apical region in some types [3]. The adhesive luting could improve post retention [4].

However, the establishment of effective bonding within the root canal is still a challenge; consequently, debonding of the post and the endodontic lesion has been described as the predominant failure mode of the restoration [5]. This failure is attributed to intrinsic difficulties in relation to correct handling of adhesive systems in narrow and deep root canal space with limited access and visibility [6] and high configuration factor (C-factor) within the confined space of root canal [7] that can even exceed 200. This unfavorable clinical situation results in polymerization shrinkage stress that exceeds cement‒dentin bond strength, causing debonding [7]. Furthermore, a thick and heavy secondary smear layer is formed during post space preparation [8, 9]. This could jeopardize prognosis of root canal therapy and interfere with bonding efficacy of adhesive systems to root dentin. The bonding ability is affected by different adhesive approaches and irrigants used for removal of the smear layer [10].

Etch-and-rinse (ER) adhesive systems, as the most clinically proven method, have been speculated to benefit from dissolving the smear layer in the root canal [11]. Self-etching (SE) systems have been introduced to overcome and simplify the sensitive bonding procedures. However, there is a concern regarding the permeation ability of self-etch adhesives through the thick smear layer, creating a hybridized smear layer and a true hybrid layer [11–14]. Therefore, the role of post space irrigant is of greater interest for SE systems [15] and optimal treatment of the smear layer should be considered. Sodium hypochlorite (NaOCl) and ethylene diamine tetra acetic acid (EDTA) are widely used as root canal irrigants. The bond strength of fiber posts to radicular dentin could be affected by various irrigants differently, depending on different types of resin cement [8, 10, 14, 16–18]. Different results have been reported after comparisons have been made between the push-out bond strengths of fiber posts using SE and ER systems with different irrigants [8, 12, 13, 19, 20]. It is worth noting that two types of adhesives with different compositions were used in the reported studies for comparison of two bonding approaches.

Recently, universal adhesives (UAs) have been introduced that can be used in ER, SE or selective enamel etching approach, depending on different clinical conditions and clinicians’ preferences [21]. These new adhesives consist of only a single bottle, with simple and short application times becoming gradually popular in adhesive procedures. UAs are essentially single-bottle SE adhesives that are designed for ER application without compromising the bonding efficacy. Different findings have been reported on the performance of UAs in ER or SE approach on dentin, which might be material-dependent [21, 22].

However, till date, the reported studies have been conducted on coronal dentin and no study has been performed on bonding performance of UAs for post cementation in root canal space using dual-cured resin cements.

The use of only one adhesive (possibly, a UA) for both coronal and root dentin bonding could simplify bonding procedures for post cementation and subsequent coronal build-up. Therefore, the aim of this study was to evaluate bonding effectiveness of All-Bond Universal/resin cement with different adhesive approaches employed to lute FRC posts (Trasluma Post Iso #100, Bisco, Schaumburg, IL, USA) in three levels of the root canal. The two-step ER, One-Step Plus (Bisco, USA) and two-step SE, Clearfil SE Bond (Kuraray, Osaka, Japan) were also assessed as control adhesives. The null hypotheses tested were that 1) push-out bond strength of All-Bond Universal to intraradicular dentin with different adhesive approaches relative to the irrigant used does not differ from the respective control adhesives; 2) the bond strengths are not affected by three root canal levels (apical, middle and coronal).

## Materials and methods

Fifty-six sound human maxillary central incisors with similar size and anatomic shape (round root canal) and straight roots without cracks were selected and stored in 0.5% chloramine T solution at 4°C until used for the purpose of this in vitro study. They were used following informed consent from patients and approval of the research protocol by the Ethical Committee of Shiraz University of Medical Sciences (Shiraz, Iran). The roots were separated from the crowns in a uniform length of 15 mm, using a water-cooled diamond saw (D&Z, Berlin, Germany). The roots were endodontically instrumented at a working length of 1 mm from the apex with K-files (Dentsply, Maillefer, Ballaigues, Switzerland) up to #45 with saline solution and sodium hypochlorite and obturated using gutta-percha and AH26 sealer (Dentsply, Caulk, Milford, Germany). The filled roots were coronally sealed using light-cured Fuji II LC glass-ionomer (GC, Tokyo, Japan). The specimens were stored in water for one week for the complete setting. Afterward, post spaces were prepared to a standardized depth of 10 mm using respective drills from the post manufacturer by the same operator. Cleanliness of the root canal walls and the remaining 4 mm of gutta-percha at the root end for apical seal were confirmed by radiographs.

The fiber posts were tried in the canals for a passive fit in the prepared depth. Post surfaces were cleaned with ethanol, air-dried and then coated with the adhesive and light-cured, according to manufacturer’s recommendation.

The specimens were randomly divided into seven groups (n = 8) based on irrigation/adhesive procedures as follows. Group 1 (ER control, OS/H): The post space was first irrigated with 5 mL of sodium hypochlorite (1% NaOCl, ChloraXiD, PPH Cerkamed, StalowaWola, Polska), followed by 5 mL of distilled water (W). Then, the post space was acid-etched with phosphoric acid for 15 s using an endodontic syringe with endodontic tips, water-rinsed for 30 s, and gently dried with absorbent paper points. One-Step Plus was applied using endodontic brushes. Group 2 (SE control, CSE/H): The post space was irrigated with NaOCl as described for group 1and Clearfil SE Bond was applied. Group 3 (CSE/ED): The post space was irrigated with 5 mL of 17% ethylene diamine tetra acetic acid (EDTA) for 60 s, followed by 5 mL of W. After slight drying, CSE was applied. Group 4 (AB-Er/H): After NaOCl irrigation and acid etching as described in group 1, All-Bond Universal (AB) was applied. Group 5 (AB-Se/H): After NaOCl/W irrigation and slight drying, AB was applied. Group 6 (AB-Se/ED): After EDTA/W irrigation, AB was applied. Group 7 (AB-Se/W): AB was applied after W irrigation. Description of the seven groups is shown in Table 1. All the adhesives were applied according to the respective manufacturer’s instructions (Table 2) by the same operator. The mixed cement (Duo-link, Bisco) was applied to the post surface and to the post space using elongation tips attached to the automixed, supplied by the manufacturer. The post was immediately seated with a slight vibratory motion and held under finger pressure. After removing the excess cement, light polymerization was carried out for 60 s at a light intensity of 600 mW/cm^2^ using a light-curing unit (VIP Junior, Bisco, Schaumburg, IL, USA) according to manufacturer’s instructions. The specimens were stored in distilled water at 37°C for one week.

**Table 1:** Description of the study groups.

Groups	Code	Adhesive category	Irrigant + adhesive steps
1	OS/H	Etch-and-rinse	NaOCl + acid etching + One-Step Plus
2	CSE/H	Self-etch	NaOCl + Clearfil SE Bond
3	CSE/ED	Self-etch	EDTA + Clearfil SE Bond
4	AB-Er/H	Multi-mode	NaOCl + acid etching + All-Bond Universal
5	AB-Se/H	Multi-mode	NaOCl + All-Bond Universal
6	AB-Se/ED	Multi-mode	EDTA + All-Bond Universal
7	AB-Se/W	Multi-mode	Water + All-Bond Universal

**Table 2:** Composition and application mode of the used adhesives system.

Adhesive system/Manufacturer (Lot No.)	Adhesive type	Application procedure	Composition
Clearfil SE Bond/ Kuraray, Osaka Japan (Primer: 01226A, Bond: 01851A)	Self-Etch (SE)	Apply primer to root canal and leave in place for 20 s. Dry with the air stream to evaporate volatile ingredients. Apply Bond to the root canal and then create a uniform film using air stream. Light polymerize for 20 s.	Primer: water, MDP, HEMA, camphorquinone, hydrophilic dimethacrylate Bond: MDP, Bis-GMA, HEMA, Camphorquinone hydrophobic dimethacrylate N, N. diethanol-toluidine.
One-StepPlus/Bisco, Schaumburg, IL, USA (1600000604)	Etch-and-Rinse (ER)	Apply etchant for 15 s. Rinse thoroughly. Remove excess water with paper points. Apply adhesive in 2 coats with agitating movements for 10 s. Blot the canal dry with paper points until the paper returns dry from the canal. Air-dry after 10 s. light cure for 20s.	Biphenyl dimethacrylate 2-hydroxyethyl methacrylate. Acetone amine, photoinitiator, dented glass
All-Bond Universal/Bisco (1500002859)	Self-Etch (SE) Etch-and-Rinse (ER)	Apply two, separate coats of adhesive, scrubbing with the micro-brush for 10–15 s per coat. Blot the canal dry with paper points until the paper returns dry from the canal. Evaporate excess solvent by thoroughly air-drying with an air syringe for at least 10 s. Light polymerize for 20 s.Apply etchant for 15 s. Rinse thoroughly. Remove excess water with paper points. Apply adhesive as for the self-etch mode.	10-MDP phosphate monomer, bis-GMA, HEMA, ethanol, water, initiators

### Push-out test and failure mode analysis

**Fig 1:**
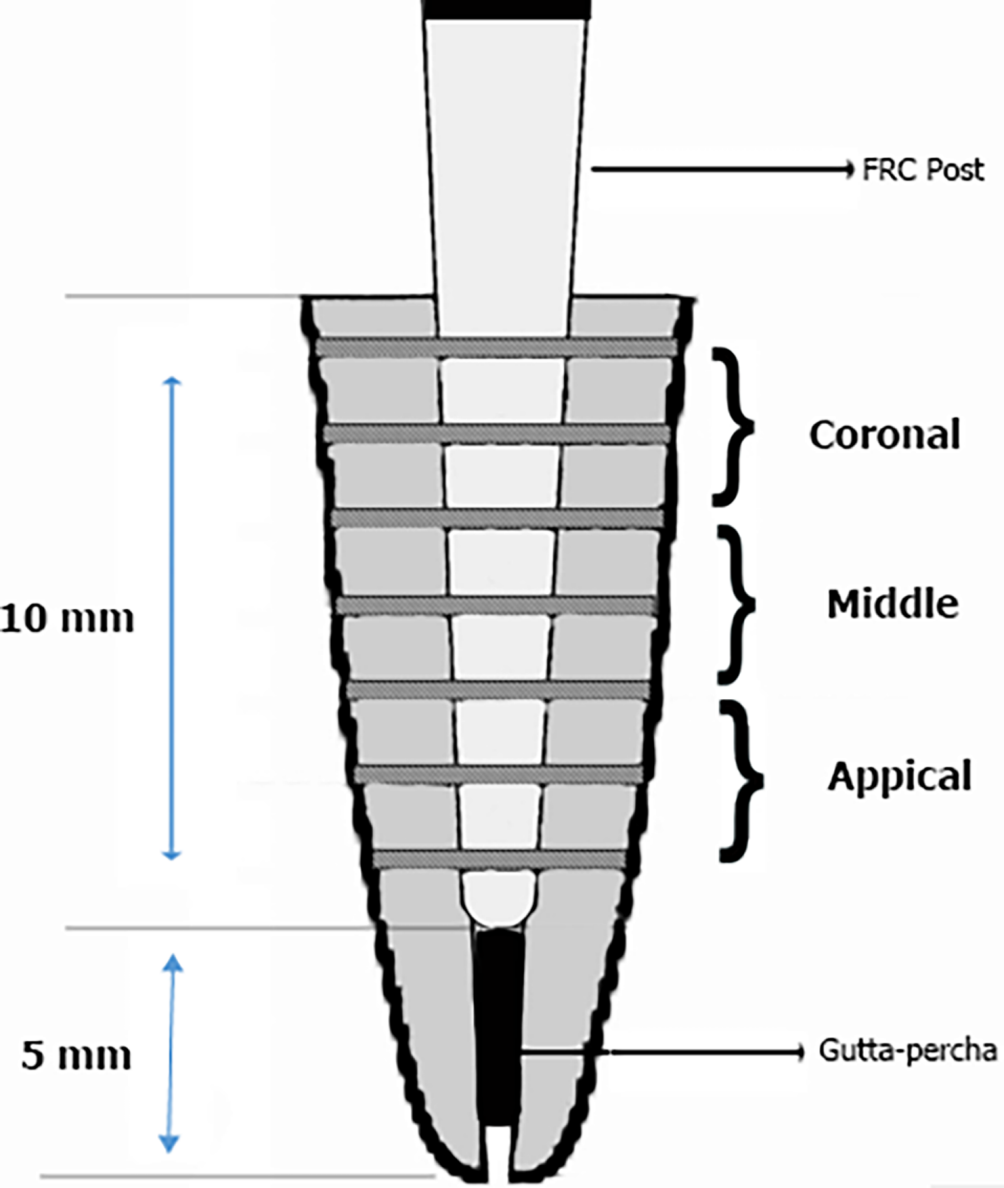
Schematic representation of the two coronal slices, two middle slices and two apical slices obtained from fiber post cemented in root canal space.

The bonded roots were sectioned into seven 1-mm-thick slices by using a slow-speed cutting machine (Mecatome T201 A, Persi, Grenoble, France). For each root, two slices from each root region (apical, middle and coronal) were obtained (Fig 1). Therefore, the sample size was 48 for each group (16 for each root region in each group). The first coronal slice was not included. The slices were subjected to a compressive load in a universal testing machine (Zwick, Roell, Ulm, Germany) at 0.5 mm/min at the center of the post in an apico-coronal direction with no contact with the root dentin or cement until the shear stresses along the bonded interface dislodged the post. With regard to the tapered design of the post, the loading was performed using three punch tip diameters. The load at debonding in Newton (N) was divided by the bonded interface area (mm^2^) and the bond strength was recorded in MPa. The bonded area was calculated through the formula π (R+r) [h^2^+(R-r) 2]^0.5^, where R and r represent the coronal and the apical post radii, respectively, and h is the thickness of the slice.

All the debonded specimens were assessed under a stereomicroscope (Carl Zeiss Inc, Oberkochen, Germany) at ×40 and categorized as follows: (1) cohesive failure in dentin; (2) cohesive failure in the cement; (3) adhesive failure between the cement and the dentin; (4) adhesive failure between the cement and the post; and (5) mixed failures consisting of a combination of two or more failure modes.

### Scanning Electron Microscope (SEM) evaluation of the adhesive interface

One additional bonded root from each group was prepared for SEM analysis. After sectioning the roots, the sections were treated with 37% phosphoric acid for 15 seconds and 5% NaOCl for 2 minutes. They were then dried in a desiccator for 24 h, sputter-coated with gold and observed under a scanning electron microscope (KYKY, EM3200, China).

### Statistical analysis

Data were statistically analyzed using two-way ANOVA and Tukey multiple comparisons (α = 0.05).

## Results

Table 3 shows the mean push-out bond strengths (PBS) and standard deviations (SD) in MPa. The PBS was significantly affected by irrigation/adhesive protocol (p<0.001) and the root region (p<0.001). However, the interaction of these two factors was not significant (p>0.05), meaning that different adhesive protocols had similar behavior with respect to root region variable (regional variation).

**Table 3:** Push-out bond strength values (mean ± standard deviation in MPa) of the tested groups.

Groups	Root region			
	Coronal	Middle	Apical	Total
OS/H	13.26 (2.9)	11.70 (2.7)	10.99 (2.4)	11.98 (2.7)
CSE/H	14.52 (3.3)	11.09 (3.5)	8.11 (1.9)	11.25 (3.9)
CSE/ED	16.98 (1.5)	13.64 (2)	11.36 (1.8)	13.99 (2.9)
AB-Er/H	14.16 (2.4)	12.8 (2.4)	11.98 (2.7)	12.99 (2.5)
AB-Se/H	11.64 (3.8)	10.0 (3.3)	8.84 (3)	10.07 (3.5)
AB-Se/ED	17.52 (3.6)	15.19 (4.6)	13.42 (2.5)	15.38 (3.9)
AB-Se/W	13.0 (2.1)	11.5 (2.6)	9.1 (1.8)	11.22 (2.7)
Total	14.44 (3.4)A	12.28 (3.4)B	10.55 (2.8)C	12.42 (3.6)

Means with different letters show statistically significant differences between root regions (p<0.05).

The results of HSD Tukey tests of all the multiple comparisons among the three root regions were significant (p≤0.001), with a reduction in PBS values from the cervical to the apical region. When various adhesive protocols were compared regardless of the root region, the highest PBS was recorded in the AB-Se/ED group (15.38±3.9). This PBS value was significantly higher than that in the other groups (p≤0.02), but not significantly higher than that in the CSE/ED group (13.99±2.9, p = 0.47). The latter group yielded a significantly higher PBS than that in CSE/H (11.25±3.9, p = 0.004).

The lowest PBS was obtained for AB-Se/H (10.17±3.5), which was not significantly different from OS/H (11.98±2.7, p = 0.17), CSE/H (p = 0.76) and AB-Se/W (11.22±2.7, P = 0.77). AB-Se/H had a significantly lower BS compared to that of AB-Er/H (12.99±2.6, p = 0.003). AB-Er/H had a PBS comparable to AB-Se/W and the control ER group, OS/H (p>0.05) (Table 4). The results of failure mode assessment revealed that the majority of failures were mixed failures in all the groups, except for the AB-Se/H group in which the bond failures were mainly adhesive failure at dentin‒cement interface.

**Table 4:** Results of multiple comparisons by Tukey test.

Groups	2	3	4	5	6	7
1	0.950	0.087	0.809	0,170	*P<0.001	0.943
2	-	*0.004	0.203	0.762	*P<0.001	1.00
3		-	0.809	*P<0.001	0.477	*0.003
4			-	*0.003	*0.020	0.193
5				-	*P<0.001	0.777
6					-	*P<0.001

1, OS/H; 2, CSE/H; 3, CSE/ED; 4, ABEr/H; 5, ABSe/H; 6, ABSe/ED; 7, ABSe/W

*P<0.05 indicates a statistically significant difference.

### SEM observations

**Fig 2:**
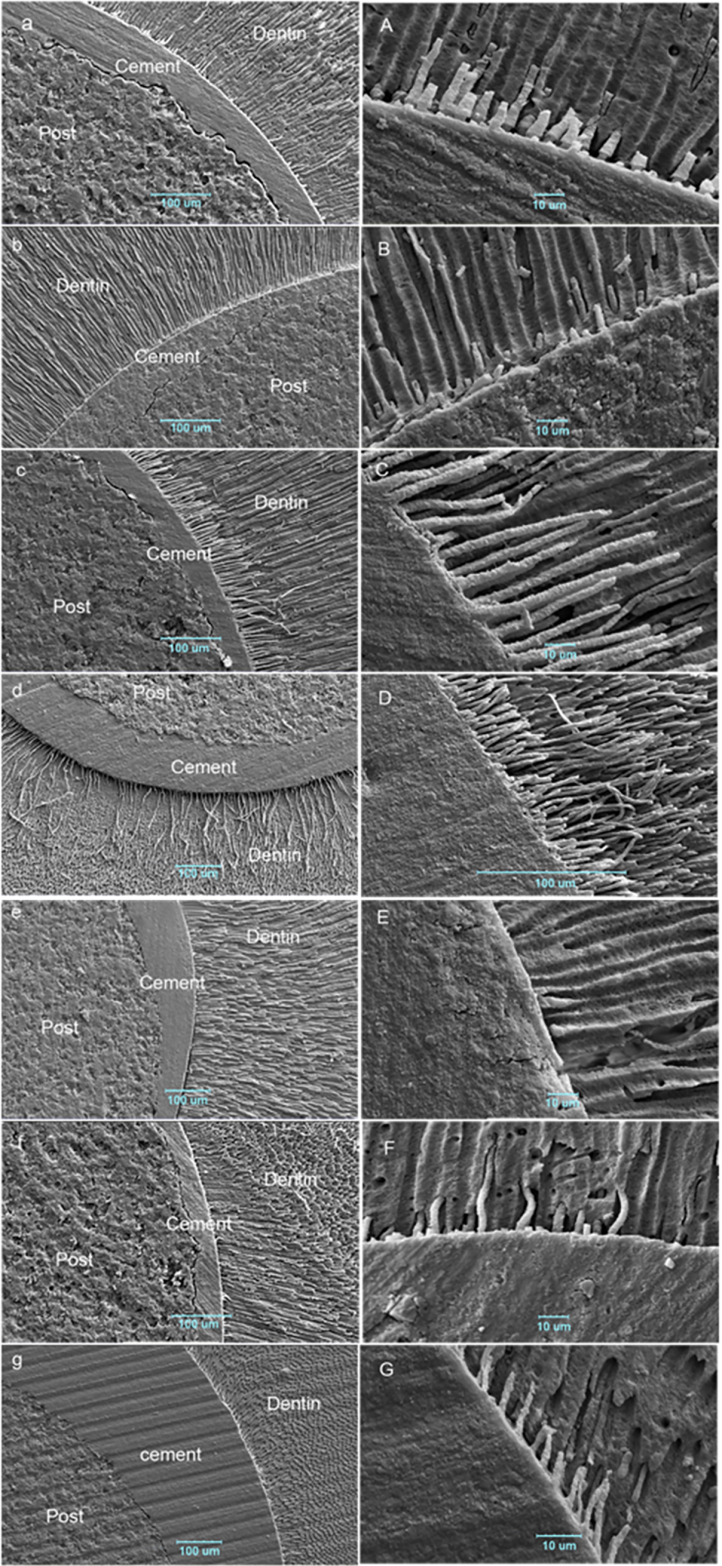
Representative SEM images of the adhesive interface in the seven study groups at low and high magnifications. a and A: OS/H, intimate adaptation with resin tag formation was observed; b and B: CSE/H, no gap was seen with few short and some fractured resin tags; c and C: CSE/ED, a well-adapted interface was found with homogenous compact resin tags; d and D: AB-Er/H, a well-formed interface was detected with numerous long resin tags; e, E: AB-Se/H, gap formation was revealed with no resin tags; f and F: AB-Se/ED, an adapted interface was detected with uneven resin tag formation; g and G: AB-Se/W, good adaptation was seen with resin tag formation.

**Fig 3:**
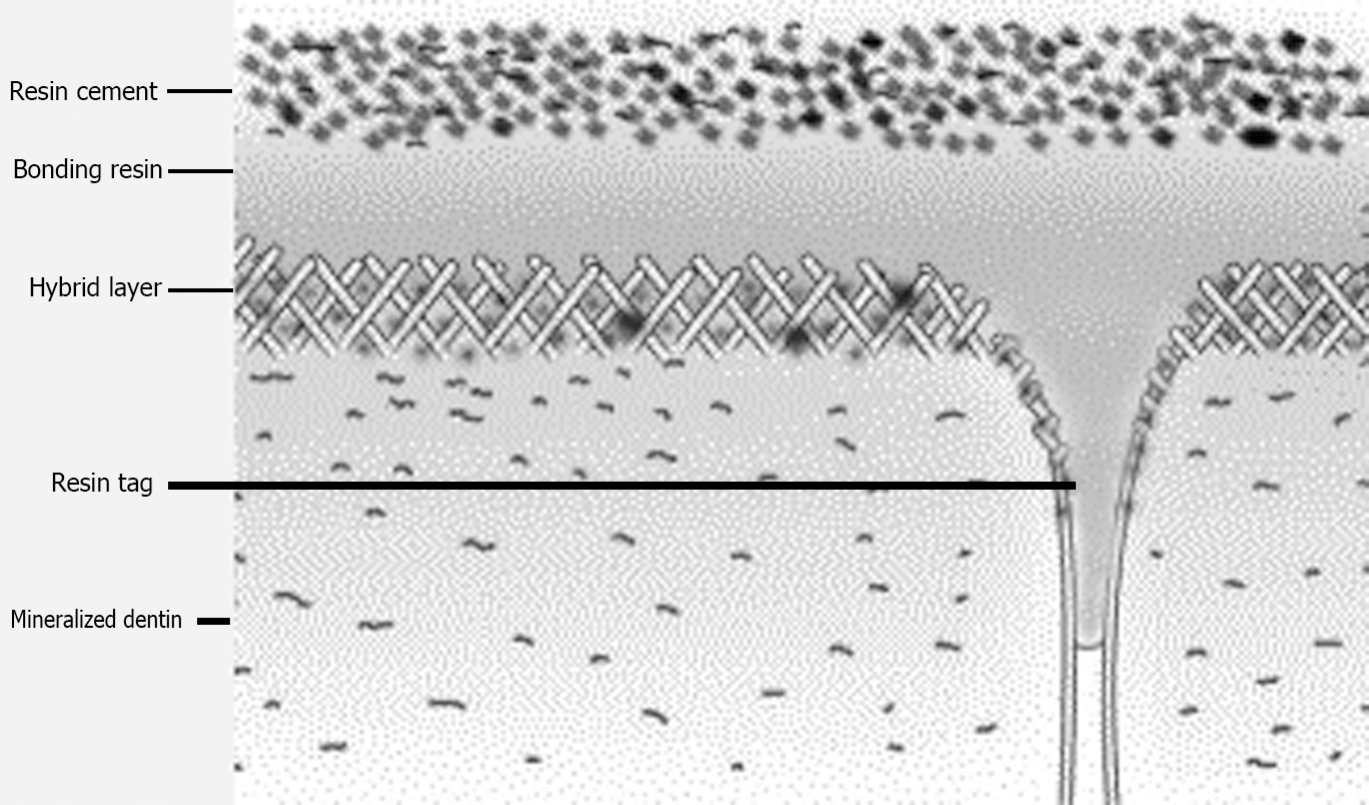
Schematic representation showing the adhesion mechanism at the adhesive interface.

The morphological images of the root dentin-adhesive interface with different irrigant/adhesive protocols under SEM are shown in Fig 2 (a-g in low magnification and A-G in high magnification). These images were randomly selected for each group. In all the groups, intimate adaptation was seen along with resin tags in different numbers and lengths, except for the AB-Se/H group in which gap formed at the interface with no sign of resin tags. In the CSE/H group, a few short and fractured resin tags were observed. The values of measured length of the resin tags are presented in Table 5. In some specimens, a separation between the post and the cement was observed, which could be attributed to shrinkage during SEM processing. This was observed in specimens of CSE/H, OS/H, and AB-Se/ED groups. A schematic representation of the adhesion mechanism at the adhesive interface is shown in Fig 3.

**Table 5:** The length of resin tags (in μm) formed at the adhesive interface for the tested groups.

Groups	The length of resin tags
OS/H	10–25
CSE/H	5–10
CSE/ED	34–72
AB-Er/H	70–149
AB-Se/H	0
AB-Se/ED	8–29
AB-Se/W	8–16

## Discussion

The present study evaluated the bonding performance of a UA, All-Bond Universal, in differently prepared root canal spaces at three levels with the SE and ER modes. In light of our findings, the null hypotheses were rejected because of significant effects of irrigation/adhesive approaches and the root levels (apical, middle and coronal).

UAs have pH values ranging between 2 and 3; only AB with a pH of 3.2 is claimed to be compatible with self- and dual-cured resin cements without any separate activator [23]. However, the relatively low acidity of AB could limit penetration through thick endodontic smear layer to impregnate the root dentin. The manufacturer (Bisco) recommends this adhesive for luting endodontic posts with optional acid etching of post space. This procedure was supported by our results because of similar PBS of this group and the OS control group.

Some studies reported better performance of ER adhesive cement than that of SE cement in terms of bonding to root dentin [13, 19]. This was attributed to less effectiveness of SE cement in etching the thick smear layer [19]. However, some reports demonstrated higher PBS of SE cement compared to that of ER cement. The technique sensitivity of wet-bonding used in ER resin cements might explain lower bonding ability of ER systems [20, 24, 25].

Sodium hypochlorite (NaOCl) is a halogenated compound with an effective antimicrobial capacity and low surface tension [26]. The latter might improve dentin wettability and flow into the root canal [27]. NaOCl was used, alone in different concentrations (1% to 5.25%), as a common endodontic irrigant for dissolving organic tissue with no ability to dissolve inorganic component of the thick smear layer [10, 26]. NaOCl combined with EDTA is used to simultaneously deproteinize and demineralize the root dentin. The irrigants used in post space were reported to affect bonding ability of various resin cement types differently [10]. The combination of NaOCl with EDTA was suggested to mostly remove the smear layer and smear plugs prior to an SE adhesive in root space [15]. However, this protocol was reported to have an insignificant negative effect on another SE and a positive effect on an ER system [8]. These authors believed that acid etching used in ER adhesives subsequent to EDTA led to excessive demineralization/erosion of root dentin, compromising bonding durability [11]. Similarly, an adverse effect of this protocol on the optimal adhesiveness of ER adhesive systems was reported by Bitter et al due to the altered chemical structure of dentin [10]. 1% NaOCl with passive ultrasonic irrigation followed by distilled water was found to be effective for the ER cement [10]. Therefore in the current study, 1% NaOCl and then distilled water were applied to all the groups before adhesive cementation. With respect to the important role of treatment of the smear layer for SE cements, EDTA was used for CSE and UA in two experimental groups instead of NaOCl. This approach revealed significantly beneficial effects on their bonding ability so that use of EDTA prior to AB in the SE mode resulted in the highest BS, which was not different from the CSE/ED group. The efficacy of EDTA for removal of the smear layer and debris along the post space was previously indicated [14, 28].

The lower effect of NaOCl compared to EDTA on the performance of the SE adhesives used might be attributed to the lower efficacy of NaOCl for the removal of the smear layer. This was documented in an SEM study by Gu et al [14]. Although in their study the smear layer removal was more effective in the NaOCl group than in the control group, a significantly lower PBS was reported for the NaOCl group (5.25% for 15 seconds) with ED primer/Panavia F 2.0 in the prepared root canal space [14]. However, a lower PBS obtained in this study for AB-Se/H than AB-Se/W was not statistically significant. The chemical structure alteration of collagens and/or oxidizing/polymerization inhibitory effect of NaOCl might contribute to the reported bond strength reduction [29, 30]. The adverse effect of NaOCl (5.25% for 1 min) on PBS of fiber posts depended on adhesive type. It reduced PBS of SE cement, while it slightly increased PBS of ER adhesive cement. Acid etching after NaOCl may remove the remaining NaOCl in superficial dentin [31]. This step or water rinsing after NaOCl treatment was speculated to limit the negative (polymerization inhibitory) effect of NaOCl [10, 15, 31]. This application sequence is an important factor. NaOCl irrigation for 10 min after acid etching decreased PBS of ER adhesive cement [32]. However, the latter sequence and long application time of NaOCl is not clinically applicable. Therefore, in this study, NaOCl was used before acid etching in OS/H and AB-Er/H groups to mimic clinical situations. An adverse effect of NaOCl on post luting with SE cement was confirmed by Martinho et al [33]. They compared the effect of three irrigants on PBS of fiber posts cemented with Fuuturabond DC and concluded that, contrary to NaOCl, saline solution and chlorhexidine had no effect. Also, supplementary root dentin pretreatment with ultrasound and Nd: YAG laser did not improve PBS in the three irrigants [33]. The relatively short application time (15 s) could limit the oxidizing effect [8]; the concentration of NaOCl might be an important factor for the negative effects. Also, various experimental designs used might result in reported convergent findings. Hayashi et al reported a lower shear BS of an SE cement to root dentin for EDTA compared to NaOCl (5% for 15 s). They warned against the removal of the smear layer via EDTA for adhesion of the cement [8]. However, that study was carried out on flattened inner surfaces of coronal half of roots prepared by silicon carbide paper, possibly resulting in the lower thickness of the smear layer than in root canal space prepared for fiber post luting [8].

Contrary to phosphoric acid, EDTA as a mild chelating agent enables selective removal of hydroxyapatite and non-collagenous protein while preserving the native collagen structure with interfibrillar mineral. Consequently, the higher stability and lower sensitivity to water content of this substrate along with residual mineral could provide a favorable penetrable bonding substrate for SE adhesives containing functional monomers [14, 34]. These could explain the higher bonding ability of AB in the SE mode for the EDTA group compared to the ER mode, whereas it was significantly better in the ER mode than in the SE mode in the hypochlorite group. The two modes were comparable when distilled water was used in the SE mode. The latter result could support the "universal/multi-adhesive approach" concept behind new UAs so that PBS to root dentin did not differ in the ER and SE modes, although the SE mode benefited considerably from EDTA conditioning approach. Some authors found that the ER mode resulted in a higher bonding ability of AB to the coronal dentin than that of the SE mode [22, 35–37]. The suggested possible explanation was inability of AB to effectively act as a self-etch primer for dentin through the smear layer due to its ultra-mild acidity (pH = 3.2) [37]. However, in line with our results, the results of studies by Wagner et al and Chen et al indicated no significant differences in dentin BS of AB between the ER and SE modes [38, 39]. Active brushing of AB in two layers was reported as a possible factor responsible for the reported result by these authors [38]. The beneficial effect of this application mode on BS of one-step SE adhesives was previously demonstrated [40, 41] through increased monomer penetration and solvent evaporation, and subsequent chemical interaction of acidic monomers with dentin and increased polymerization efficacy [42]. These factors might be more relevant with AB that contains more solvent (30‒60 wt%) and the functional monomer 10-MDP in the root canal space with clinical difficulties in achieving sufficient adhesion. This monomer is capable of forming a protective stable nano-layer and a stronger phase at the adhesive interface, increasing mechanical strength and durability of the interface [43]. Phosphoric acid etching ensures removal of the smear layer and smear plugs and resin impregnation into demineralized dentin, facilitating the formation of longer resin tags and a thicker hybrid layer [21]. The length of resin tags was reported to reach 50 μm for AB on coronal dentin with a thin smear layer (created by SiC paper) [38]. This occurrence was also observed on the root dentin that was prepared for post insertion in our SEM analysis of the interface. This demonstrated the mentioned efficacy of acid etching in thicker smear layer in root canal space. Therefore numerous resin tags up to 149 μm length were achieved in the AB-Er/H group compared to fewer and shorter resin tags (8‒16 μm) in the AB-Se/W group. Despite this difference in the interface micromorphology, a similar PBS was obtained. The role of resin tag formation in bonding is controversial. Resin tags were reported to contribute about 30% to the total bond strength [44]. In SEM studies, resin tags demonstrated a mechanism for adhesive bonding to root dentin [44, 45]. However, the absence of a clear relation between resin tag formation and BS was reported for SE adhesives [46] and recently for UAs in coronal dentin [38]. Bitter et al found no correlation between morphological characteristics of the adhesive interface and bonding different adhesive cements to root dentin [25]. Nevertheless, the lowest PBS of AB-Se/H group was well reflected in our SEM observation with gap formation between the cement and dentin without any resin tags. This observation was also supported by failure mode analysis after debonding; therefore, the predominant failure mode of this group was adhesive between the root dentin and the cement. Furthermore, the beneficial effect of EDTA on PBS of two SE adhesives was indicated in SEM finding in a better way for CSE. The long homogenous, compacted resin tags were pointed. The separate resin layer in CSE adhesive might contribute to this well-formed interface. The primer and resin component of CSE contained MDP. According to these observations and PBS results, the slight micromechanical interlocking associated with the chemical interaction in AB-Se/W group seemed to be as effective as only prominent micromechanical retention in ER mode. However, the role of chemical bonding capacity of MDP in the ER approach with no residual calcium is not clear. This ability might be involved in the significantly higher PBS obtained in AB-Se/ED group. Some authors confirmed this beneficial property following the higher PBS of SE MDP-based cements compared to ER ones when 2.5% NaOCl or chlorhexidine was used as an irrigant [17].

Long-term storage and thermal fatigue might better predict the clinical behavior of UAs [37]. However, it was reported that PBS of fiber posts to root dentin was not affected by thermocycling [47] and mechanical cycling [48, 49]. Nevertheless, further long-term investigations in the presence of thermal and load cycling could be conducted to provide valuable information regarding the bonding longevity of UAs under difficult conditions found in the root canal space.

## Conclusion

Considering the limitations of this study, bonding effectiveness of AB in ER and SE modes was similar to the respective control adhesives (OS and CSE). Use of irrigants influenced the adhesive performance of the SE mode; it benefitted from EDTA, resulting in better performance of the SE mode than the ER mode. On the contrary, the adverse effect of NaOCl leads to the lower performance of the SE mode compared to the ER mode.

## Supporting information

S1 File Measurements of push-out bond strength values (MPa) of the tested groups. https://doi.org/10.17026/dans-zph-hw9x.
(XLSX)
